# Navigating PRKCSH’s impact on cancer: from N-linked glycosylation to death pathway and anti-tumor immunity

**DOI:** 10.3389/fonc.2024.1378694

**Published:** 2024-03-20

**Authors:** Ratchada Cressey, Moe Thi Thi Han, Worapong Khaodee, Guo Xiyuan, Yuan Qing

**Affiliations:** ^1^ Department of Medical Technology, Faculty of Associated Medical Sciences, Chiang Mai University, Chiang Mai, Thailand; ^2^ Cancer Research Unit, Department of Medical Technology, Faculty of Associated Medical Sciences, Chiang Mai University, Chiang Mai, Thailand; ^3^ Public Experimental Technology Center School of Basic Medical Sciences, Southwest Medical University, Luzhou, China

**Keywords:** PRKCSH, glucosidase II beta subunit (GluIIβ), N-linked glycosylation, death pathway, anti-tumor immunity, cancer, apoptosis, autophagy

## Abstract

PRKCSH, also known as Glucosidase II beta subunit (GluIIβ), is a crucial component of the endoplasmic reticulum (ER) quality control system for N-linked glycosylation, essential for identifying and eliminating misfolded proteins. Glucosidase II consists of the catalytic alpha subunit (GluIIα) and the regulatory beta subunit (GluIIβ), ensuring proper protein folding and release from the ER. The induction of PRKCSH in cancer and its interaction with various cellular components suggest broader roles beyond its previously known functions. Mutations in the *PRKCSH* gene are linked to autosomal dominant polycystic liver disease (ADPLD). Alternative splicing generates distinct PRKCSH isoforms, which can influence processes like epithelial-mesenchymal transition (EMT) and the proliferation of lung cancer cells. PRKCSH’s involvement in cancer is multifaceted, impacting cell growth, metastasis, and response to growth factors. Additionally, PRKCSH orchestrates cell death programs, affecting both autophagy and apoptosis. Its role in facilitating N-linked glycoprotein release from the ER is hypothesized to assist cancer cells in managing increased demand and ER stress. Moreover, PRKCSH modulates anti-tumor immunity, with its suppression augmenting NK cell and T cell activity, promising enhanced cancer therapy. PRKCSH’s diverse functions, including regulation of IGF1R and IRE1α, implicate it as a therapeutic target and biomarker in cancer immunotherapy. However, targeting its glucosidase II activity alone may not fully counteract its effects, suggesting broader mechanisms in cancer development. Further investigations are needed to elucidate PRKCSH’s precise role and validate its therapeutic potential in cancer treatment.

## Introduction

PRKCSH (Protein kinase C substrate 80K-H), a key component of the endoplasmic reticulum quality control system for N-linked glycosylation, plays a vital role in recognizing and eliminating misfolded proteins (1). As the beta subunit (GluIIβ) of the glucosidase II enzyme, its interactions with other proteins and involvement in cellular processes make PRKCSH indispensable for maintaining protein homeostasis. Dysregulation of PRKCSH has been linked to various diseases, including cancer.

**Figure 1:**
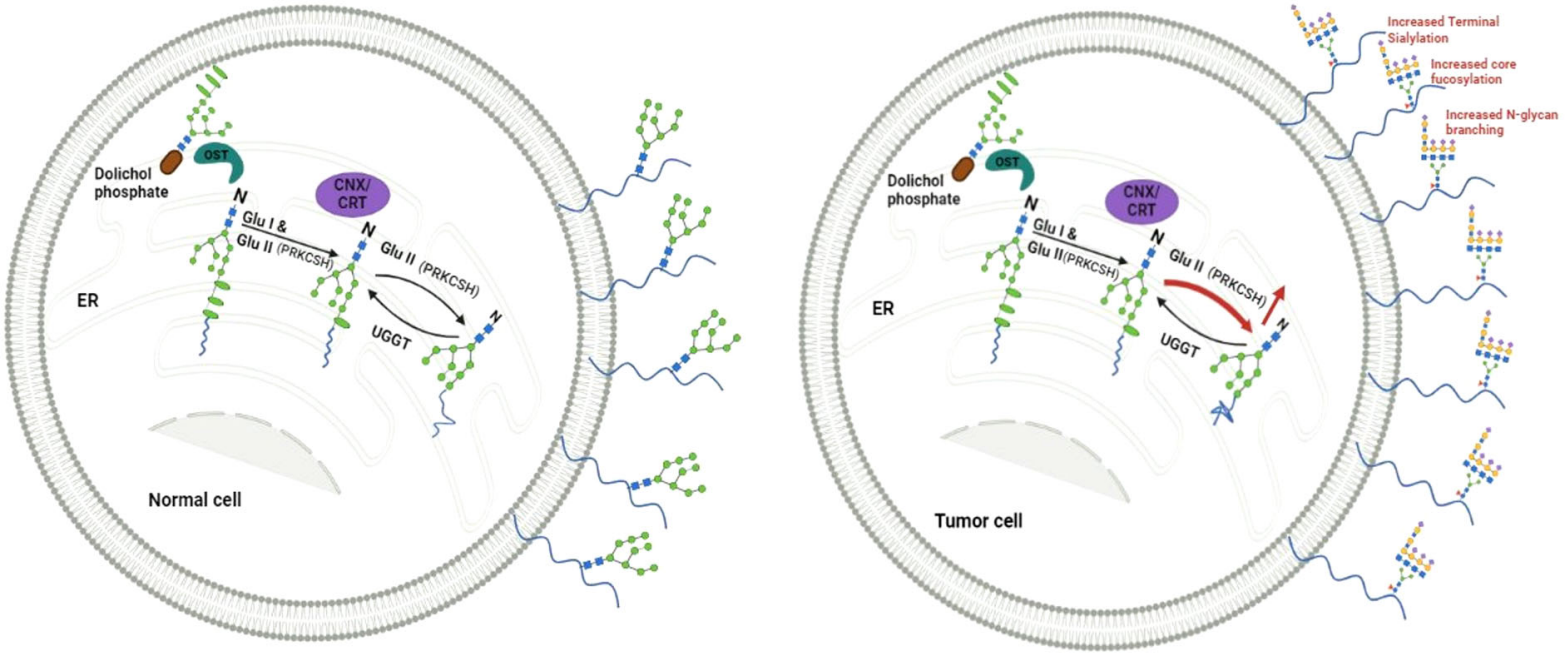
Depicts a proposed model illustrating the equilibrium between Glucosidase II (GluII), with the beta subunit encoded from PRKCSH, and UGGT (UDP-glucose:glycoprotein glucosyltransferase) in normal cells, maintaining a steady level of N-linked glycoproteins on the cell surface. Conversely, elevated levels of Glucosidase II in cancer cells facilitate meeting the heightened demand for N-linked glycoproteins, including those with altered structures.

In the endoplasmic reticulum (ER), as part of N-linked glycosylation, proteins being synthesized are modified by attaching a pre-made oligosaccharide. This oligosaccharide contains 2 N-acetylglucosamines, 9 mannoses, and 3 glucoses (2). This glycosylation is believed to enhance the hydrophilicity of unstructured proteins. The glycosylated protein chains then enter a specialized chaperone system involving calnexin and calreticulin (3). To access the calnexin/calreticulin system, it is crucial to eliminate outer glucose residues. This process is accomplished by the consecutive activity of two enzymes, namely glucosidase I and glucosidase II (4). The balance between glucosidase II and UDP-glucose: glycoprotein glucosyltransferase-1 (UGGT) activities become crucial for ER protein quality control. While glucosidase II facilitates proper protein folding and release from the ER, UGGT can add back a terminal glucose to promote re-association in case of misfolded proteins, prolonging their residence in the ER (**Figure 1**).

Glucosidase II comprises a catalytic alpha subunit (GluIIα) and a regulatory beta subunit (GluIIβ). In-depth research on glucosidase II’s alpha subunit has primarily focused on maturation, stabilization, localization, and N-glycan recognition. However, questions persist regarding the less-explored beta subunit. This includes its induction in cancer (5, 6) and in rat progenitor cell lines responding to GDNF (glial cell-derived neurotrophic factor), its non-correlation with GluIIα expression regulation (7), and its intriguing interaction with the 3’UTR of the R1-subunit mRNA of the N-methyl-D-aspartate (NMDA) receptor in mouse fetal cortical neurons (8). Mutations in the *PRKCSH* gene have been associated with the inheritance pattern of autosomal dominant polycystic liver disease (ADPLD) (9), and numerous alternatively spliced transcript variants have been documented (10, 11), resulting in the production of different isoforms of PRKCSH. These discoveries imply broader roles for PRKCSH. Gaining a comprehensive understanding of the intricacies surrounding PRKCSH may offer valuable insights into cellular glycosylation events, developmental processes, and potential implications in cancer. This review highlights the intricate interplay between PRKCSH (GluIIβ) and cancer, emphasizing its significance in various cellular processes and its potential as a key player in cancer-related mechanisms.

## Characteristic of PRKCSH protein

PRKCSH firstly isolated by Sakai et al. in 1989 (12), located on chromosome 19p13.2. It is also known as Glucosidase II beta subunit (GluIIβ). PRKCSH has various aliases including 80K-H, hepatocystin, PCLD (polycystic liver disease), PLD1 (phospholipase D1, phosphatidylcholine-specific), G19P1, advanced glycation end product receptor-2 (AGE-R2), and Protein kinase C substrate 60.1 kDa protein heavy chain. The protein exhibits a complex structure with several distinctive features, including four potential protein kinase C phosphorylation sites, an EF-hand domain for calcium binding, and a significant glutamic acid repeat (13). Notably, it contains a C-terminal HDEL sequence indicative of endoplasmic reticulum retention and proline-rich motifs (PXXP), potentially involved in binding affinity to the SH3 domain of GRB-2 (growth factor receptor-bound protein 2). PRKCSH has a polar distribution of cysteine residues suggesting the presence of multiple intra- and/or intermolecular disulfide bridges. This multifaceted nature of PRKCSH hints its involvement in various cellular processes and interactions, contributing to its role in health and disease.

PRKCSH has been identified as an advanced glycation end product (AGE) receptor on the plasma membrane of various human cell types. Its role involves facilitating cell activation, chemotaxis, and secretion (14). Its involvement extends to the regulation of inositol 1,4,5-trisphosphate receptors (IP3Rs) in COS-7 cells and hippocampal neurons, influencing Ca^2+^ release from the endoplasmic reticulum (15). Additionally, it interacts with the epithelial Ca^2+^ channel TRPV5 (Transient Receptor Potential Cation Channel Subfamily V Member 5) in HEK293 cells (16), plays a role in fibroblast growth factor (FGF) signaling in human breast cancer cells and rat myoblast cells (5, 17), and is implicated in intracellular vesicle transport in CHO (18). Collectively, PRKCSH exhibits a wide array of functions across various subcellular locations.

## *PRKCSH* gene mutations

Mutations in the *PRKCSH* gene have been identified in association with autosomal dominant polycystic liver disease (ADPLD). ADPLD is characterized by the formation of numerous cysts spread throughout the liver tissue. In advanced stages, the presence of large cysts can result in heightened pressure within the abdomen, leading to disability in specific individuals. The connection between PRKCSH and ADPLD was initially identified in 2000 (9). Subsequent investigations revealed that many ADPLD patients with mutations in PRKCSH exhibit a clustering of mutations in specific domains (19). The results of mutation analysis showed that frameshift mutations were the most common, comprising 31.4% of the total mutations (11 out of 35). Following behind were splice mutations and nonsense mutations, each making up 22.9% of the mutations. Combined, loss-of-function mutations, which include nonsense, frameshift, and splice mutations, accounted for 77.1% of all reported mutations.

Patients carrying mutant PRKCSH typically exhibit a spectrum of PCLD severity, ranging from mild cases with a positive family history to more severe cases without a family history (19). This diverse clinical presentation underscores the complexity of the genetic factors influencing the manifestation and progression of ADPLD associated with PRKCSH mutations.

## Alternative splicing of *PRKCSH* gene

Multiple alternatively spliced transcript variants, generating distinct isoforms of PRKCSH, have been reported (10, 11). Examination of cDNA clones and partial genomic DNA sequencing in T lymphocytes has unveiled alternative splicing in PRKCSH gene products (10). Bioinformatic analysis further identified numerous transcript variants, including PRKCSH-1 and PRKCSH-2, as prominent alternative splice transcripts with specific roles in regulating Epithelial-Mesenchymal Transition (EMT) progression (11). Silica exposure induces varied alternative splice variants in silicosis patients, indicating altered expression of PRKCSH-1 and PRKCSH-2 in response to silica insults. The loss of PRKCSH-2 promotes lung cancer cell proliferation by modulating the EMT pathway. These findings highlight the dynamic nature of PRKCSH isoforms in response to environmental insult and their significant implications in cancer progression.

## Potential role of PRKCSH in cancer development

The role of PRKCSH in oncogenesis was initially suggested by an investigation of somatic mutations in the GAG stretch of the *PRKCSH* gene in gastric cancer, and a significant association was found between *PRKCSH* GAG mutations and a high level of microsatellite instability (MSI-H) (20). Increased PRKCSH protein expression was observed in breast cancers (5) and lung cancer (6). Notably, PRKCSH exhibited the ability to translocate to the nucleus in response to acidic fibroblast growth factor (aFGF or FGF-1) treatment, leading to heightened cell growth and invasion (5). Furthermore, studies conducted in Swiss 3T3 cells and MRC-5 fibroblasts revealed that PRKCSH undergoes phosphorylation upon treatment with basic fibroblast growth factor (bFGF or FGF-2). The phosphorylated form of PRKCSH was then translocated from the cytosol to the membrane fraction (13). PRKCSH’s role extends beyond solid tumors; it has been reported to regulate hematopoietic stem cell (HSC) function, with high expression predicting poor survival in acute myeloid leukemia (AML) patients (21). We also reported that knockout of *PRKCSH* gene inhibited growth and metastatic potential of lung cancer cells by inhibiting receptor tyrosine kinase activities (22).

The comprehensive investigation into the involvement of PRKCSH in cancer development using the complete data sets of human tumor tissues of The Cancer Genome Atlas (TCGA), as detailed by Shin et al. (23), revealed a significant upregulation of PRKCSH expression across various cancer tissues. These cancers encompassed esophageal carcinoma, glioblastoma, liver hepatocellular carcinoma, lymphoid neoplasm, thymoma, pancreatic adenocarcinoma, skin cutaneous melanoma and stomach adenocarcinoma. Moreover, elevated expression of PRKCSH was observed in cancer tissues from the liver, colon, gastric, breast, and lung. High PRKCSH expression was notably associated with extrahepatic metastasis, advanced TNM stage, and a poor survival rate among patients (23). Bioinformatics analysis of PRKCSH expression in cancer databases to study its role in lung cancer revealed that higher PRKCSH expression was linked to worse outcomes for lung cancer patients (24). PRKCSH expression level was negatively associated with STAT6 levels in lung cancer tissues. Further investigation revealed that suppression of PRKCSH led to G2/M cell cycle arrest of lung cancer cells treated with Nano ZnO, which hypothesized to mediate through the translocation of STAT6 to nucleus and activate p53 (24). These findings collectively suggest the existence of growth factor/PRKCSH signaling pathways, highlighting its potential pivotal role in the initiation and progression of cancer.

## PRKCSH and the regulation of death program in cancer cells

Cell death programs are essential for balancing cell survival and elimination, and their dysregulation is a hallmark of cancer. The subsequent discussion explores the impact of PRKCSH on these programs, shedding light on its potential significance in cancer development. PRKCSH’s involvement in cell death pathways was initially demonstrated by Yang et al. (25) in autophagy, a cellular process responsible for degrading and recycling cellular components. Knocking down PRKCSH induced autophagy mediated through an mTOR-dependent pathway. Reduced glucosidase II activity upon PRKCSH knockdown leads to autophagy activation, independent of IP3R-mediated calcium flux. Notably, the unfolded protein response (UPR) pathway remains unaffected by PRKCSH deficiency (25).

The involvement of PRKCSH in apoptosis cell death program was first reported in plant cells (26). This study found that PRKCSH could signal the cell to either undergo autophagy for stress-damaged parts clean up or initiate apoptosis for self-destruction under excessive stress. Mild ER stress in rice cells led to a decrease in PRKCSH levels, promoting autophagy. In contrast, severe ER stress caused PRKCSH to be cleaved by caspase-3-like activity, resulting in a fragment that initiated apoptosis. Under severe stress, PRKCSH formed a complex with heat shock proteins GRP94 (glucose-regulated protein 94) and HSP40 (heat shock protein 40), eventually cleaved by caspase-3-like activity at the DEYD109S site. The C-terminal cleavage product of PRKCSH further promoted caspase-3-like activity, establishing a caspase-3-amplifying feedback loop.

Our research also demonstrated PRKCSH’s involvement in mediating both autophagy and apoptosis depending on p53 status. PRKCSH suppression, either by siRNA to suppress PRKCSH expression level or selective glucosidase II inhibitor, induced autophagy in both wild-type p53 and p53-null lung carcinoma cells, while apoptosis was triggered exclusively in cells carrying wild-type p53 (27). Importantly, blocking autophagy enhanced the effectiveness of PRKCSH inhibition in killing cancer cells independently of p53 status through apoptosis emphasizing its potential as a target for cancer therapy. These findings underscore the dual role of PRKCSH in regulating autophagy and apoptosis, suggesting that the suppression of PRKCSH, in combination with autophagy, effectively directs cancer cells toward apoptosis. It is plausible that an increased level of PRKCSH may be a mechanism employed by cancer cells to suppress death pathways, explaining why the suppression of PRKCSH triggered cancer cell death.

## PRKCSH’s involvement in managing ER stress in cancer cells

As the regulatory subunit of the glucosidase II enzyme, PRKCSH assumes a critical role in facilitating the release of N-linked glycoproteins from the endoplasmic reticulum (ER), thereby mitigating ER stress. Cancer cells frequently encounter heightened ER stress due to factors like rapid cell proliferation, nutrient deprivation, and hypoxia (28). ER stress is an adaptive mechanism that helps restore balance in the ER when it is under severe environmental stress, aiding cell survival by activating the unfolded protein response (UPR). The UPR alleviates ER stress by enhancing protein folding capacity, reducing protein synthesis, and promoting the degradation of misfolded proteins (29).

In coping with these challenging conditions, it is hypothesized that the increased expression of PRKCSH assists in meeting the heightened demand for N-linked glycoproteins in cancer cells. N-linked glycoproteins, which play integral roles to various facets of cancer development, have been implicated in promoting cell growth (30). Notable examples include the Epidermal Growth Factor Receptor (EGFR) and Transforming Growth Factor-Beta (TGF-β) Receptor, which are critical in regulating cell growth. Aberrant glycosylations of these receptors are linked to increased signaling activity, contributing to uncontrolled cell proliferation in diverse cancers (31, 32). Another example is PD-L1 (Programmed Death-Ligand 1), which is an immune checkpoint molecule that suppresses anti-tumor immunity by binding to PD-1 on T cells. This interaction inhibits the activity of T cells, enabling tumor cells to evade immune surveillance. Altered N-glycosylation of PD-L1 was reported to affect its activity by influencing its stability, subcellular localization, and interaction with PD-1, potentially modulating immune checkpoint signaling and impacting anti-tumor immunity (33).

Our study revealed that suppressing PRKCSH led to a dose-dependent down-regulation of EGFR/RTK and PI3K/AKT signaling activities, resulting in significantly reduced growth rates, particularly in conditions of low nutrient availability (22). This emphasizes the critical role of PRKCSH in the cells’ ability to adapt to and survive in less favorable environments. Additionally, PRKCSH has been identified as a selective activator of the IRE1α branch of the unfolded protein response (UPR), contributing to tumor cell adaptation to stress and the initiation of tumorigenesis (23). This study demonstrated that PRKCSH enhanced the Inositol-Requiring Enzyme 1 alpha (IRE1α) signaling pathway by promoting autophosphorylation and oligomerization of IRE1α through direct interaction under ER stress. Elevated levels of PRKCSH correlated with the expression of tumor-promoting genes. The authors proposed that PRKCSH modulated glycoprotein quality control under normal conditions and activated the IRE1α-mediated stress response pathway upon ER stress through domain-specific interactions. This dual function of PRKCSH in ER protein quality control and as a regulator of IRE1α under ER stress reveals its intricate involvement in cellular adaptation and survival mechanisms.

## Anti-tumor immunity and PRKCSH

The IRE1α signaling pathway, a crucial component of the unfolded protein response (UPR) reported to be directly activated by PRKCSH (23), plays a multifaceted role in the context of anti-tumor immunity. Activation of IRE1α in cancer cells has been demonstrated to sustain microsomal prostaglandin E synthase-1 (mPGES-1) expression, leading to the production of immunosuppressive prostaglandin E2. This mechanism facilitated the advancement of NSCLC by compromising the ability of adaptive immune cells in the tumor microenvironment (TME) to effectively destroy tumor cells (34). In NSCLC, the activation of the IRE1α-XBP1 signaling pathway was associated with a poor prognosis. When the IRE1α gene was knockout from cancer cells, the progression of tumors in a mouse model of NSCLC was slowed down, leading to an extension in overall survival.

The understanding of the relationship between the IRE1α signaling pathway and anti-tumor immunity has expanded to include its influence on shaping the phenotypes and functions of immune cells, such as dendritic cells (35) and macrophages (36), crucial players in anti-tumor immune responses. Moreover, IRE1α activation in tumor cells induced the production of specific cytokines that impacted the local immune response, contributing to the modulation of anti-tumor immune activities (37). Additionally, IRE1α activity in cancer cells has been linked to the regulation of tumor immunogenicity, affecting their susceptibility to immune surveillance.

The activation of IRE1α by PRKCSH implies that inducing PRKCSH in cancer cells could influence their evasion of immunosurveillance. Our recent study, utilizing CRISPR/Cas9 to generate PRKCSH knockout A549 cells and employing transcriptomic analysis via RNA sequencing, revealed a down-regulation of genes related to the extracellular matrix (ECM), cell adhesion molecules (CAMs), and cytokine interactions (38). Gene expression validation was performed through real-time quantitative RT-PCR, and immune cell functions were assessed via co-culture assays. Notably, PRKCSH knockout A549 cells exhibited altered cell morphology and enhanced tumor lysing capability of Jurkat E6.1 T cells and peripheral blood mononuclear cells (PBMCs). Through bioinformatic analysis of TCGA (The Cancer Genome Atlas), GTEx (The Genotype-Tissue Expression), CCLE (Cancer Cell Line Encyclopedia), GSCA (Generalized Structured Component Analysis), CancerSEA (Cancer single-cell state atlas), and Cmap (Connectivity Map) databases, Wang and colleagues observed that PRKCSH expression showed an inverse relationship with the infiltration of M1 macrophages in six different cancer types. Conversely, they found a positive association between PRKCSH expression and the infiltration of M2 macrophages in twelve tumor types. It also showed negative correlations with activated mast cells in nine tumors and with the infiltration of CD4 and CD8 T cells in various tumors (39). Shin GC et al. recently showed that PRKCSH increased the stability and activation of the insulin-like growth factor 1 receptor (IGF1R) in lung cancer. This enhanced resistance to tumor necrosis factor superfamily (TNFSF), leading to tumor growth and suppressing the immune system’s ability to fight against the tumor (40). Collectively, these results suggest an augmented immune response against cancer cells in the absence of PRKCSH, highlighting its potential as a therapeutic target for improving anti-tumor immunosurveillance.

## Clinical implication and therapeutic potential of PRKCSH

**Figure 2:**
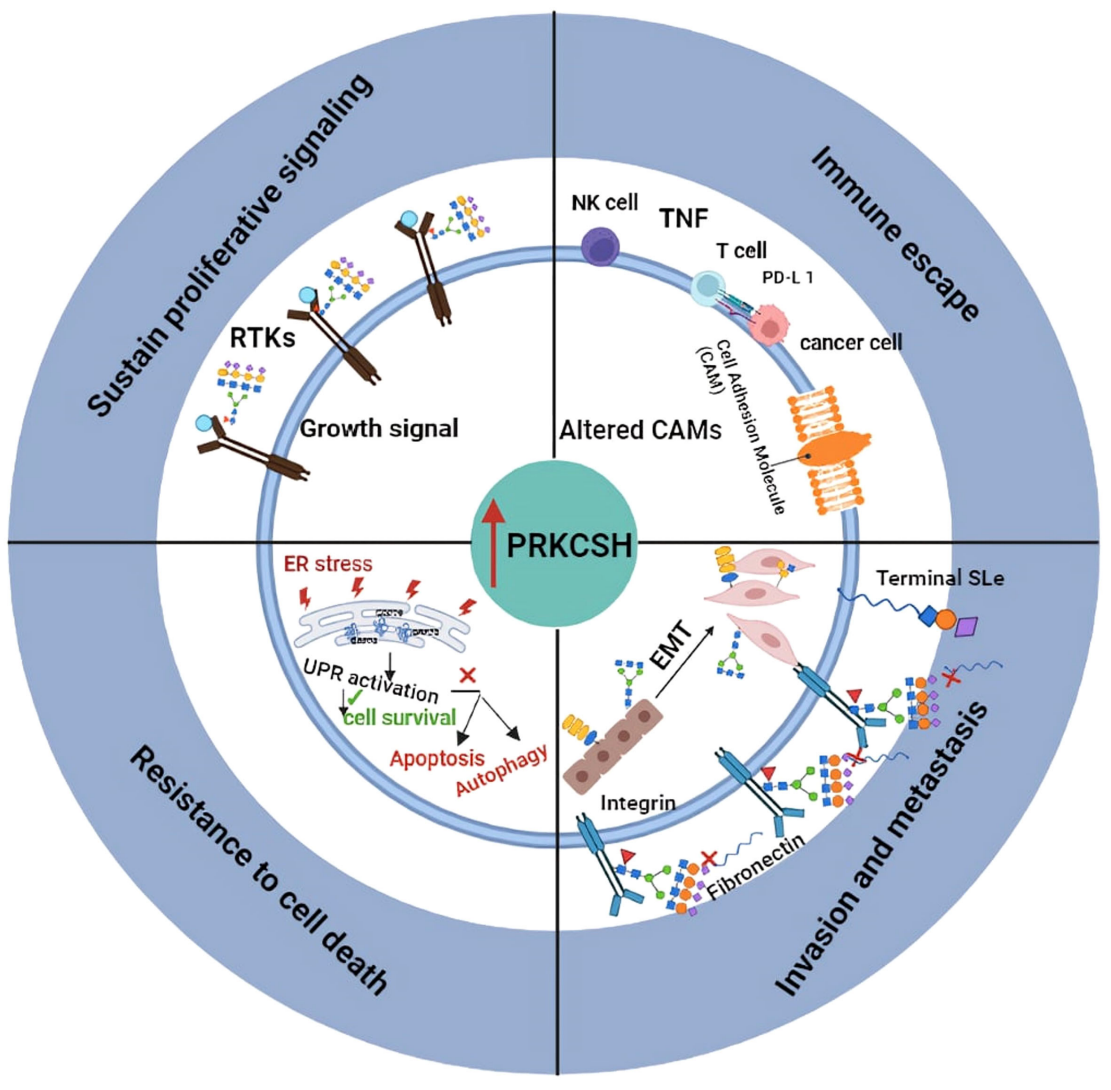
llustrates the proposed role of PRKCSH in promoting tumor growth through mechanisms including resistance to cell death, sustained proliferation signaling, promoting invasion and metastasis, as well as mediating immune escape.

PRKCSH overexpression correlates with poorer prognosis in various cancers, implicating its role in initiating and promoting tumorigenesis (**Figure 2**). Targeting PRKCSH and its associated pathways emerges as a potential therapeutic strategy for cancer management. PRKCSH stands out as a potential therapeutic target to induce cancer cell death. Downregulating PRKCSH expression or blocking the activity of glucosidase II has been documented to prompt cancer cell death through the initiation of both autophagy and apoptosis pathways. While autophagy has dual effects, inducing apoptosis is a common goal in cancer treatment. The interplay between autophagy and apoptosis in cancer cells is gaining attention, with studies suggesting simultaneous manipulation for enhanced treatment efficacy. Developing drugs that modulate both processes is an active area of research, holding promise for more effective and selective cancer therapies. PRKCSH suppression has been demonstrated to induce both autophagy and apoptosis. Although apoptosis was specific to wild-type p53 cells, blocking autophagy in PRKCSH-inhibited cells resulted in massive apoptotic cell death in both wild-type and p53-null cells. Combining PRKCSH and lysosomal inhibitors may present a novel strategy for inducing extensive cancer cell death through apoptosis, but further clinical investigations are essential for validation.

PRKCSH emerges as a dual player in cancer immunotherapy, serving as both a therapeutic target and a biomarker. Inhibiting PRKCSH could revitalize the immune response, enhancing NK cell-based cancer therapy by disrupting IGF1R stability and activation, thus overcoming TNFSF resistance. Additionally, PRKCSH’s regulation of IRE1α and cell adhesion molecules like PD-L1 underscores its potential as a target and marker for augmenting T cell responses against cancer. Nevertheless, rigorous clinical validation in cancer patients is imperative to substantiate and refine this novel cancer treatment paradigm.

While PRKCSH shows promise as a potential therapeutic target, an important question arises: Is simply suppressing glucosidase II enzyme activity enough to counteract PRKCSH’s effects? Previous studies using gene manipulation or specific glucosidase II inhibitors indicate that while inhibiting glucosidase II activity aligns with blocking PRKCSH-mediated cell death pathways, it doesn’t fully address PRKCSH’s broader functions. Specifically, PRKCSH mediates IRE1α activation through direct interaction but not through glucosidase II enzyme activity. This disparity, along with inconsistent correlations between PRKCSH (GluIIß) expression and GluIIα, suggests that PRKCSH may contribute to cancer development through mechanisms beyond its enzyme activity. Further investigation is needed to understand PRKCSH’s intricate roles in cancer development, highlighting the importance of a comprehensive therapeutic approach.

## Author contributions

RC: Conceptualization, Funding acquisition, Supervision, Writing – original draft, Writing – review & editing. MH: Conceptualization, Visualization, Writing – original draft, Writing – review & editing. WK: Conceptualization, Writing – original draft, Writing – review & editing. GX: Conceptualization, Writing – original draft, Writing – review & editing. YQ: Writing – original draft, Writing – review & editing.
